# Continuous negative extrathoracic pressure combined with high-frequency oscillation improves oxygenation with less impact on blood pressure than high-frequency oscillation alone in a rabbit model of surfactant depletion

**DOI:** 10.1186/1475-925X-6-40

**Published:** 2007-10-31

**Authors:** Sachie Naito, Takehiko Hiroma, Tomohiko Nakamura

**Affiliations:** 1 Division of Neonatology, Nagano Children's Hospital, Nagano, Japan

## Abstract

**Background:**

Negative air pressure ventilation has been used to maintain adequate functional residual capacity in patients with chronic muscular disease and to decrease transpulmonary pressure and improve cardiac output during right heart surgery. High-frequency oscillation (HFO) exerts beneficial effects on gas exchange in neonates with acute respiratory failure. We examined whether continuous negative extrathoracic pressure (CNEP) combined with HFO would be effective for treating acute respiratory failure in an animal model.

**Methods:**

The effects of CNEP combined with HFO on pulmonary gas exchange and circulation were examined in a surfactant-depleted rabbit model. After induction of severe lung injury by repeated saline lung lavage, 18 adult white Japanese rabbits were randomly assigned to 3 groups: Group 1, CNEP (extra thoracic negative pressure, -10 cmH_2_O) with HFO (mean airway pressure (MAP), 10 cmH_2_O); Group 2, HFO (MAP, 10 cmH_2_O); and Group 3, HFO (MAP, 15 cmH_2_O). Physiological and blood gas data were compared among groups using analysis of variance.

**Results:**

Group 1 showed significantly higher oxygenation than Group 2, and the same oxygenation with significantly higher mean blood pressure compared to Group 3.

**Conclusion:**

Adequate CNEP combined with HFO improves oxygenation with less impact on blood pressure than high-frequency oscillation alone in an animal model of respiratory failure.

## Background

Continuous negative extrathoracic pressure (CNEP) applied around the chest was been shown to be efficacious in the treatment of respiratory failure in infants [1-5]. CNEP can produce increased functional residual capacity and may lead to increased cardiac output by increasing cerebral venous return and decreasing pulmonary vascular resistance [6,7]. However, wide use of this technique has not been seen in the neonatal field, as creating negative pressure around the fragile chest wall is difficult in neonates.

High-frequency oscillation (HFO) has been shown to prevent both acute and chronic lung injury in neonatal management. Specifically, HFO has been shown to reduce the incidence of chronic lung disease in very low birth weight infants [8-10]. Studies of surfactant deficiency in animal models have demonstrated that volume recruitment is one of the important lung protective strategies during HFO [11,12].

In the present study, we hypothesized that CNEP combined with HFO would offer greater improvements in oxygenation than HFO alone in a rabbit model of surfactant depletion.

## Materials and methods

### Animal model

The study protocol was approved by the Institutional Animal Care and Committee of Nagano Children's Hospital, Nagano, Japan. Eighteen adult white Japanese rabbits weighing 2.0–2.5 kg were used for this study. All animals were premedicated by intramuscular administration of ketamine (10 ml/kg) and xyladine (5 mg/kg). The peripheral ear vein was cannulated using a 24-gauge angiocatheter for intravenous anesthesia and infusion of medication. Animals were placed in a supine position during the entire study period. A 3.5-Fr endotracheal tube without cuff (Mallinckrodt, St. Louis, Missouri, USA) was inserted into the trachea and tied to prevent gas leak. The carotid artery was cannulated using a 22-gauge angiocatheter and connected to a blood pressure monitor (Polygraph System; Nihon Koden, Tokyo, Japan) to monitor arterial blood pressure and heart rate, and to obtain arterial blood samples for blood gas analysis. Anesthesia was provided by continuous intravenous infusion of ketamine (5 mg/kg/h) and paralysis was maintained using pancuronium (0.1 mg/kg/h). Mechanical ventilation was performed using a time-cycled, pressure-limited ventilator (Humming II; Metran, Saitama, Japan). Animals were administered 10% glucose in 0.45% saline solution at 3 ml/kg/h throughout the study period without any colloid or catecholamine.

### Measurements

Oxygen saturation, heart rate and blood pressure were monitored continuously using a pulse oximeter (Nihon Koden, Tokyo, Japan). Tidal volume (Vt) was measured intermittently using a low-dead space hot-wire pneumotachograph (Aivision Laminar Flow Meter LFM-317; Metabo, Lausanne, Switzerland). Arterial blood gas samples were analyzed intermittently (0, 30, 60, 90 and 120 min). Blood pressure, heart rate and ventilator settings were recorded before and after lung injury and at 30-min intervals during the 120-min study period.

### Experimental protocol

After obtaining baseline measurements, acute respiratory failure was induced by repeated lung lavage with aliquots of 30 ml/kg of warmed normal saline. Lavage was considered adequate if PaO_2 _was <80 mmHg by 15 min after last lavage with the following ventilator settings: FiO_2 _1.0 at a respiratory rate of 30 breaths/min with positive end expiratory pressure (PEEP) of 5 cmH_2_O; peak inspiratory pressure (PIP) to maintain Vt of 15 ml/kg; and inspiratory time of 1.0 s. To induce severe and stable lung injury, animals were ventilated mechanically for 60 min at the above settings.

To determine the adequate CNEP level combined with HFO (mean airway pressure (MAP), 10 cmH_2_O) in our study, we conducted a preliminary examination of oxygenation at each CNEP level (extra thoracic negative pressures: -5 cmH_2_O; -10 cmH_2_O; and -15 cmH_2_O) combined with HFO. CNEP (-5 cmH_2_O) combined with HFO showed no change in oxygenation compared with HFO alone. CNEP combined with HFO (MAP, 10 cmH_2_O) showed the same oxygenation level at -10 cmH_2_O or -15 cmH_2_O.

From these preliminary results, we decided to use CNEP (-10 cmH_2_O) in our experimental protocol. Animals were randomly allocated to 3 therapy groups. Group 1 used CNEP (extra thoracic negative pressure -10 cmH_2_O) with HFO (MAP 10 cmH_2_O). CNEP (RTX; Medivent, London, UK) settings were as follows: CNEP at -10 cmH_2_O and neonatal size selected for the cuirass. HFO settings were as follows: MAP at 10 cmH_2_O and pressure amplitude adjusted to maintain PaCO_2 _between 35 and 55 mmHg at a frequency of 15 Hz. Group 2 used HFO alone at MAP 10 cmH_2_O, and Group 3 used HFO alone at MAP 15 cmH_2_O.

### Statistical Analysis

All results are expressed as mean ± standard deviation, and were compared using analysis of variance (ANOVA) for repeated measures with Scheffé's test. Values of p < 0.05 were considered statistically significant.

## Results

**Figure 1:**
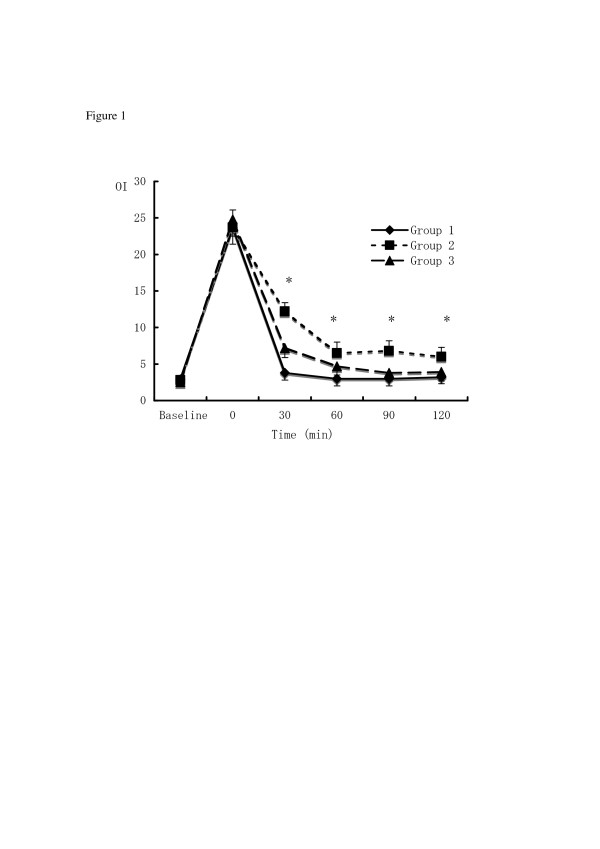
**Changes in oxygen index (OI) in experiments**. (Diamond) Group 1: CNEP (-10 cmH_2_O) with low-MAP (10 cmH_2_O) HFO. (Circle) Group 2: Low-MAP (10 cmH_2_O) HFO. (Square) Group 3: High-MAP (15 cmH_2_O) HFO. *p < 0.05 Groups 1, 3 vs. Group 2.

**Figure 2:**
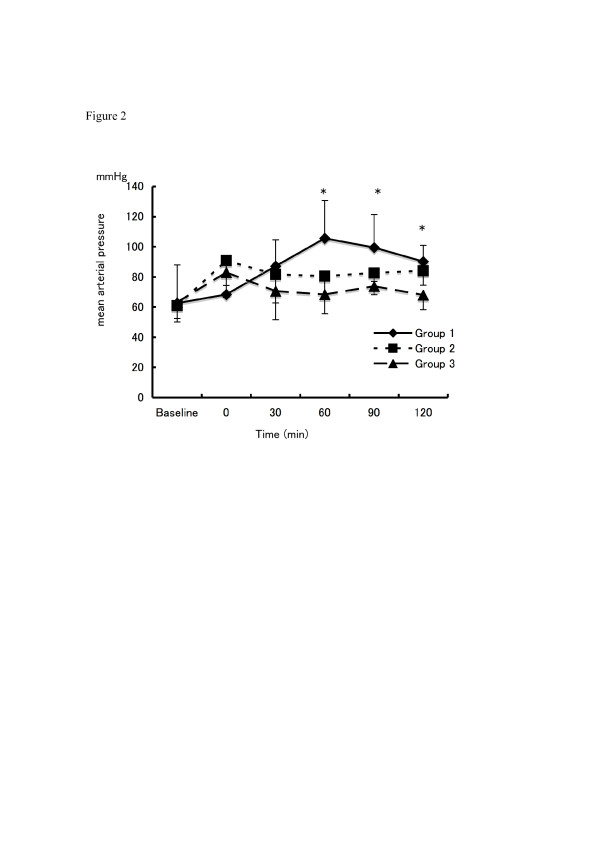
**Changes in mean arterial pressure**. (Diamond) Group 1: CNEP (-10 cmH_2_O) with low-MAP (10 cmH_2_O) HFO. (Circle) Group 2: Low-MAP (10 cmH_2_O) HFO. (Square) Group 3: High-MAP (15 cmH_2_O) HFO. *p < 0.05 Group 1 vs. Group 3.

Baseline and post-injury data were similar in all 3 groups. Changes in PaO_2 _over time are shown in Figure 1. In Group 1 (-10 cmH_2_O CNEP with HFO; MAP 10 cmH_2_O), PaO_2 _increased after starting CNEP and was significantly higher than in Group 2 (HFO; MAP, 15 cmH_2_O) (p < 0.05). Group 3 (HFO; MAP 15 cmH_2_O) displayed similar PaO_2 _to Group 1. Changes in MAP during the observation period are shown in Figure 2. Mean arterial pressure was significantly lower in Group 3 than in Group 1 (p < 0.05) throughout the experimental period.

## Discussion

PEEP is generally accepted to increase transpulmonary pressure, thus increasing lung volume and reopening some previously collapsed lung units. An alternative approach to increasing transpulmonary pressure, and thus lung volume, is represented by application of negative pressure around the chest. Randomized trials to assess the benefits of CNEP and standard care in preterm infants have been described [3]. Telford et al. reported long-term outcomes after neonatal CNEP [5]. They showed that death or sever disability was equally distributed between CNEP group and standard treatment group. Full IQ did not differ significantly between groups, but mean performance IQ was higher in the CNEP group. CNEP was also useful in more mature infants with other types of respiratory failure [1,2]. In the treatment of acute lung injury, application of CNEP increased transpulmonary pressure, thus achieving improved lung function similar to that obtained with PEEP. As opposed to PEEP, which increases intrathoracic pressure, CNEP increases transpulmonary pressure by decreasing intrathoracic pressure, rather than by increasing airway pressure. CNEP has favorable effects on permeability and hydrostatic pulmonary edema [6,7]. In a sheep model inoculated with *Pseudomonas *bacteria, CNEP decreased hydrostatic filtration pressure and lung lymph flow [13]. In dogs with pulmonary edema induced by oleic acid, CNEP increased extravascular lung water volume, but did not change central blood volume [14]. Shekerdemian et al. reported that CNEP improved cardiac output in children after cardiac surgery [15-17]. However, CNEP has been not widely used in neonatal intensive care unit, as extrathoracic devices are not easy to fix to the chest wall of neonates, and maintaining constant extrathoracic negative pressure is difficult.

HFO is a gentler mechanical ventilation approach with very low tidal volume and fixed mean airway pressure, which decreases the pressure swing in the peripheral airways and alveoli, and may result in a reduction of lung injury. HFO started after birth can prevent the development of chronic lung disease in very low birth weight infants at high risk for respiratory distress syndrome [8-10]. Sustained increases in MAP could induce rapid, large increases in PaO_2 _in the lungs, exhibiting some hysteresis in pressure/volume relationships [11,12]. However, higher MAP utilized during HFO could conceivably impede venous return and lead to hypotension. In neonates, this might result in intracranial hemorrhage [18].

Although the present study was limited by a lack of direct measurement of transpleural pressure and cardiac output, we showed that adequate CNEP combined with HFO results in the same level of oxygenation and significantly higher mean blood pressure compared with high MAP HFO-only groups. In neonate, high MAP HFO easily affect on circulation and need volume expander or catecholamine to keep adequate blood pressure. Although, further experiments are needed to develop a more comfortable and useful cuirass that can be adjusted to individual neonatal chest size for long-term use in human neonate, we can try this ventilator combination in neonate who has severe respiratory and circulatory failure. Based on these experimental data, we speculate that adequate CNEP might play a role as a continuous volume recruitment maneuver during HFO or change in pulmonary blood flow or increases in cardiac output. Some articles have described comparative evaluations of hemodynamic effects for CNEP and positive end-expiratory pressure [19-22], no studies appear to have shown the combined effects of CNEP and HFO on oxygenation in an animal model of lung injury. We hope to look at adequate circulation in CNEP with HFO in further experiments.

We conclude that adequate CNEP combined with HFO improves oxygenation with less impact on blood pressure than HFO alone in an animal model of surfactant depletion.
